# A Case Report of Anti-N-Methyl-D-Aspartate Receptor (NMDAR) Encephalitis

**DOI:** 10.7759/cureus.78377

**Published:** 2025-02-02

**Authors:** Sofia Mahomed Mateus, Diogo Ferreira da Silva, Beatriz Sampaio, Carolina Coelho, Raffaele Aliberti

**Affiliations:** 1 Internal Medicine, Centro Hospitalar Universitário de Lisboa Central, Lisbon, PRT

**Keywords:** acute encephalitis, anti-nmdar encephalitis, encephalitis, immune-mediated encephalitis, nmdar, non-competitive n-methyl-d-aspartate (nmda)

## Abstract

Anti-N-methyl-D-aspartate (NMDA) receptor* (*NMDAR) encephalitis is a relatively recent condition, classified as an immune-mediated disorder characterized by a complex neuropsychiatric syndrome and the presence of anti-GluN1 subunit antibodies against the NMDA receptor in cerebrospinal fluid (CSF). Although it is a rare disease, studies have identified it as one of the most common causes of autoimmune encephalitis. The pathophysiology of this condition is not yet fully understood, especially regarding its association with ovarian teratomas and other neoplasms. We present the case of a 30-year-old woman, previously healthy and independent, who developed a sudden onset of psychosis, marked emotional lability, disorganized speech, and agitation. Subsequently, she experienced severe sleep deprivation accompanied by grandiose delusions and auditory hallucinations. Due to the fluctuating nature of her symptoms and their progressive worsening, the patient required prolonged hospitalization, including admissions to intermediate and intensive care units, and underwent extensive diagnostic testing before a definitive diagnosis was made. Early diagnosis of anti-NMDAR encephalitis is crucial, as prompt and appropriate treatment can significantly reduce long-term sequelae and the risk of recurrence. This case underscores the importance of considering anti-NMDAR encephalitis in the differential diagnosis of new-onset psychiatric disorders, especially those resembling schizophrenia.

## Introduction

Anti-N-methyl-D-aspartate (NMDA) receptor (NMDAR) encephalitis is a relatively recent condition, first described in 2005 by Vitaliani et al., under the term "neoplastic encephalitis." It was described as a neuropsychiatric syndrome in four female patients with ovarian teratomas. These symptoms, in addition to psychiatric manifestations with acute onset, included seizures, memory deficits, altered consciousness, and central hypoventilation [1,2].

In 2007, the condition was further identified by Dalmau et al., who referred to it as "paraneoplastic anti-NMDAR encephalitis." In their study, 12 women aged 14-44 years developed psychiatric symptoms along with amnesia, seizures, dyskinesias, autonomic dysfunction, decreased consciousness, and central hypoventilation. Antibodies targeting the NR2B subunits of the NMDA receptor were identified in this group. It was at this point that the condition was first reported, and subsequent studies have focused on its etiology, diagnosis, and treatment [3]. Consequently, anti-NMDAR encephalitis is now defined as an immune-mediated disease characterized by a complex neuropsychiatric syndrome and the presence of anti-GluN1 subunit antibodies against the NMDA receptor in cerebrospinal fluid (CSF) [4,5].

Anti-NMDAR encephalitis is a rare disorder, with an estimated incidence of one to five cases per million people per year [4]. However, according to Barry et al., epidemiological studies suggest that it may be one of the most common causes of autoimmune encephalitis [6]. The low incidence rates in the general population may be explained by the lack of awareness of the condition among healthcare professionals, leading to underdiagnosis. Another contributing factor is the possibility of it being initially misdiagnosed as schizophrenia, as the primary psychiatric symptoms with sudden onset are similar [7]. As a result, the true incidence of anti-NMDAR encephalitis in the general population and among individuals with psychosis is still unclear [6].

According to Dalmau et al., the condition predominantly affects females, with a female-to-male ratio of 8:2, and the average age is 21 years (ranging from one to 85 years). It is most commonly associated with tumors, particularly ovarian teratomas [4]. Dalmau et al. concluded that the disease primarily affects young adults, with 95% of cases occurring in individuals under the age of 45, and a higher incidence in females. Tumor frequency varies by age and gender, with a 0-5% incidence in children under 12 years, 58% in women over 18 years (mainly teratomas), and 23% in adults over 45 years, where carcinomas are more common than teratomas [8].

The pathophysiology of anti-NMDAR encephalitis remains unclear, although studies have shown autoantibodies against the GluN1 subunit of the NMDA receptor [4,7]. As the condition primarily affects females, some authors suggest that it may be triggered by ovarian teratomas, which express NMDA receptors and expose them to the immune system, leading to the formation of antibodies. Another hypothesis is that B-cells and plasma cells producing anti-GluN1 antibodies escape immune checkpoints. Additionally, in some cases, anti-NMDA encephalitis may develop as a sequela of infections caused by herpes simplex virus (HSV) or *Toxoplasma gondii*. However, the mechanism in these cases remains uncertain [9,10].

In terms of clinical presentation, this condition is described as having several stages: prodromal, psychotic, unresponsive, hyperkinetic, and gradual recovery. During the prodromal phase, flu-like symptoms are commonly observed, lasting from five to 14 days, such as fever, headache, tremors, nausea, and vomiting. After this phase, symptoms progress to the psychotic phase, with neuropsychiatric symptoms and behavioral disturbances, including apathy, fear, depression, cognitive impairment, psychosis, and seizures, most commonly generalized as tonic-clonic seizures. In this phase, patients are often referred to psychiatric services. The unresponsive phase is characterized by a lack of response to verbal commands, pain, or visual contact. Subsequently, in the hyperkinetic phase, patients exhibit involuntary orofacial movements, dysautonomia, chewing movements, cardiac arrhythmias, labile blood pressure, and hypoventilation. After tumor removal and/or immunosuppressive therapy, patients enter the gradual recovery phase. However, not all patients experience all phases [2,9,11].

A 2019 article by Dalmau et al. provides an overview of autoimmune encephalitis and describes the diagnostic approach for anti-NMDAR encephalitis [2,4]. The diagnosis of anti-NMDAR encephalitis is considered probable if: (i) there is rapid onset (within three months) of at least four of the following groups of symptoms: abnormal behavior or cognitive dysfunction; language dysfunction (e.g., mutism or reduced speech); seizures; movement disorders, dyskinesias, or abnormal or rigid posturing; decreased level of consciousness; autonomic dysfunction or central hypoventilation; and (ii) at least one of the following laboratory findings: abnormal EEG showing focal or diffuse slow activity, disorganized activity, or delta brush; CSF with pleocytosis or oligoclonal bands. Alternatively, if three of the above symptom groups are present and a systemic teratoma is identified, the diagnosis is also considered probable. In all cases, exclusion of recent HSV encephalitis or Japanese B encephalitis is required, as these conditions can result in the recurrence of neurological symptoms. The diagnosis becomes definitive if one or more of the six symptom groups are present, along with the detection of IgG GluN1 antibodies [2,4].

Regarding treatment principles, the approach is directed toward the underlying etiology, including immunotherapy, symptomatic management, and supportive care [2,12]. If a teratoma is identified, it should be surgically removed, as early removal improves neuropsychiatric symptoms and reduces the risk of recurrence [2,13]. First-line immunotherapy should involve steroids, intravenous immunoglobulin, and plasmapheresis. Second-line treatments may include rituximab and/or cyclophosphamide [2,12-14]. Symptomatic and supportive treatment should include monitoring of vital signs, administration of antipsychotics, anticonvulsants, mechanical ventilation, and nutritional support [2]. After systemic immunotherapy, approximately 75% of patients recover or have mild sequelae. The removal of the ovarian teratoma is associated with a 23% reduction in the risk of recurrence [2].

## Case presentation

A 30-year-old single, college-educated woman with no relevant medical or family history was admitted to the psychiatric emergency department with a sudden onset psychotic episode, described as significant emotional lability, disorganized speech, and restlessness. Laboratory analysis revealed leukocytosis, leukocyturia, and unfavorable toxicology results. Due to the patient's refusal of further investigation, she was discharged against medical advice.

Three days later, the patient returned to the emergency department with sleep deprivation, fluctuating symptoms, megalomaniac ideas, and auditory hallucinations. However, there was no clinical or laboratory evidence of an infectious focus, and the EEG was normal. She was then admitted to the psychiatry department, where autonomic dysfunction with tachycardia and sweating, altered consciousness, and musical hallucinations were observed. A lumbar puncture was performed, which revealed lymphocytic pleocytosis in the CSF with 32 cells, predominantly mononuclear, proteins 52 mg/dL, glucose 66 mg/dL, and lactate dehydrogenase (LDH) <30 UI/L.

Virological studies from the CSF, including tests for HSV-1 and 2, human herpesvirus (HHV)-6 and 7, varicella-zoster virus (VZV), cytomegalovirus (CMV), Epstein-Barr virus (EBV), and enterovirus, were all negative, as well as the serologies for HIV-1 and 2 and Venereal Disease Research Laboratory (VDRL). Additionally, a CT scan of the brain showed no abnormalities. Empiric acyclovir treatment was administered for six days.

Due to worsening consciousness, the patient was transferred to the intermediate care unit one day after admission, where diagnostic workup continued. Fluctuation in consciousness, fever spikes, the onset of chewing movements, and a lack of response to verbal stimuli were noted. In the absence of clinical improvement and with suspicion of paroxysmal activity, levetiracetam was initiated as a therapeutic trial.

Regarding the etiological investigation, all complementary diagnostic tests returned negative or showed no specific abnormalities. An EEG was performed and showed normal results. In this context, a brain MRI revealed no acute or expansive focal intraparenchymal lesions but identified incipient hyperintense foci in the periventricular and bilateral frontal subcortical white matter. Additionally, chest and abdominopelvic CT scans were performed to assess for occult neoplasia, along with measurement of adenosine deaminase (ADA) levels in the CSF and testing for neurotropic viruses, including tick-borne encephalitis (TBE), Toscana, West Nile, and lymphocytic choriomeningitis (LCM). The patient's thyroid function was normal, and anti-TPO, TG, and TRAB antibodies were negative.

The autoimmune workup also returned entirely negative results. Tests for erythrocyte sedimentation rate (ESR), complement components C3 and C4, anti-double-stranded DNA (dsDNA), anti-Beta2 GP1 IgG, anti-Beta2 GP1 IgM, anticardiolipin IgM, antineutrophil cytoplasmic antibodies (ANCAs), anti-deamidated gliadin IgA, anti-deamidated gliadin IgG, anti-transglutaminase IgA, anti-transglutaminase IgG, and neuronal antibodies were all negative.

The patient's condition worsened again, and methylprednisolone 2 g was initiated for five days, along with empiric doxycycline. Despite the absence of apparent risk factors, polymerase chain reaction (PCR) testing for *Rickettsia *spp. (including both *Rickettsia conorii* and non-*conorii*) as well as Zika virus was requested and returned negative. Given the possibility of autoimmune encephalitis, immunoglobulin therapy was started; however, no clinical improvement was observed after five days.

**Figure 1:**
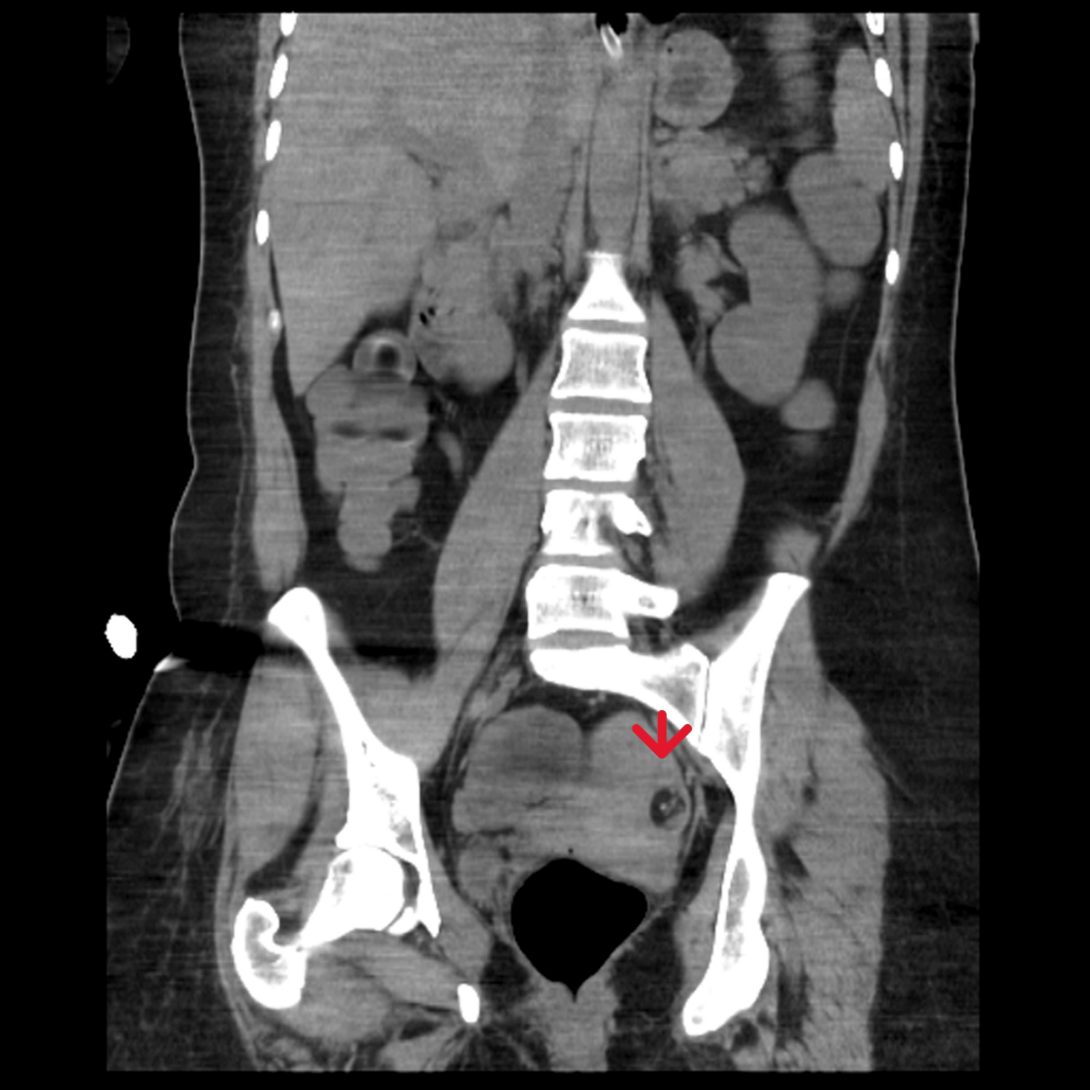
Left ovarian teratoma (arrow) measuring 18 x 18 mm, with lipomatous content and a small calcific focus inside - coronal section

**Figure 2:**
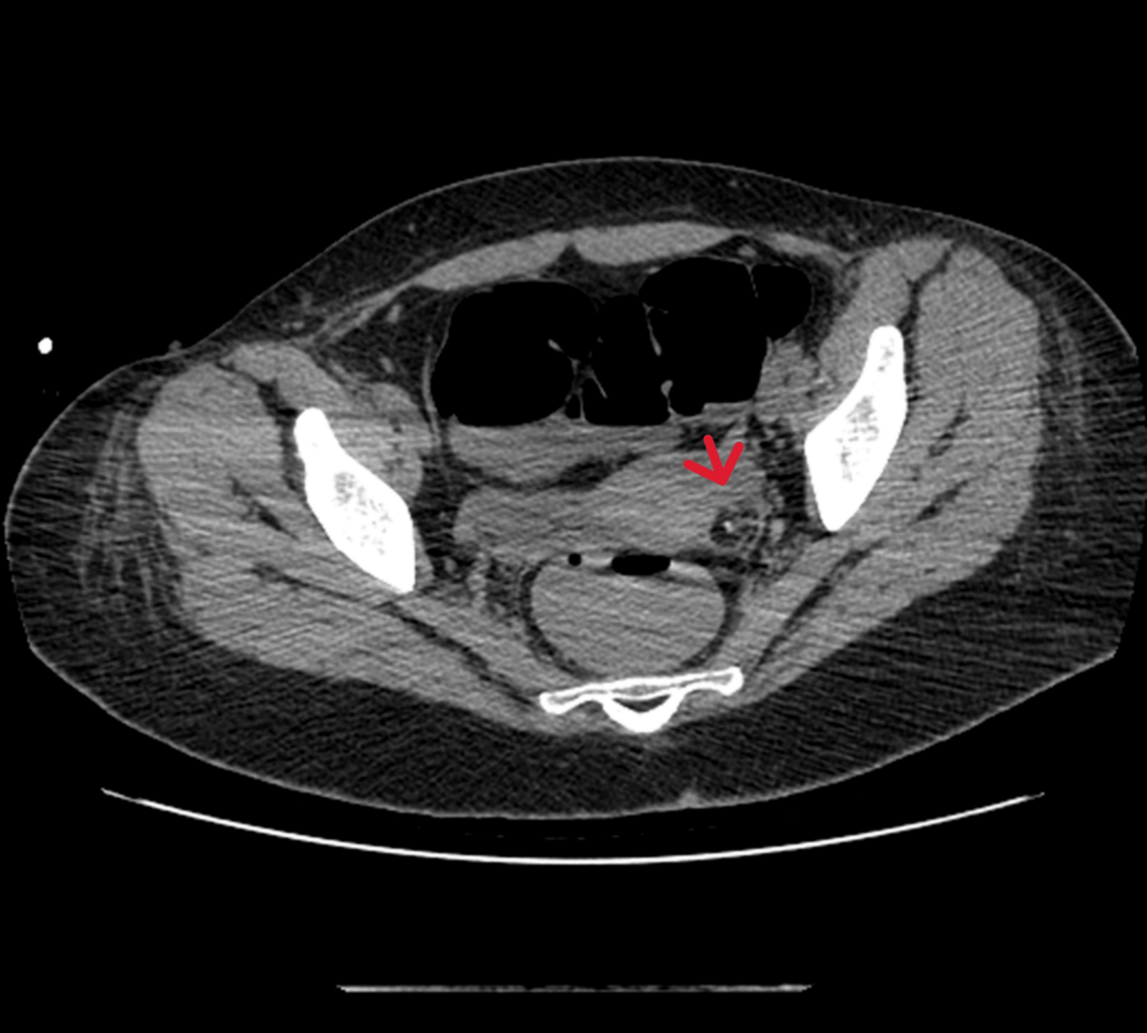
Left ovarian teratoma (arrow) measuring 18 x 18 mm, with lipomatous content and a small calcific focus inside - axial section

The patient eventually developed aspiration pneumonia, which led to admission to the intensive care unit (ICU) for invasive ventilation. A pelvic CT was repeated, revealing the presence of a left ovarian teratoma measuring 18 x 18 mm, with lipomatous content and a small calcific focus inside (Figures 1-2). Based on this finding, the patient was referred to the operating room, where an oophorectomy was performed and the mass was surgically removed. Histopathological analysis confirmed the diagnosis of a mature trigeminal teratoma of the ovary. After the surgery, the CSF was sent to an external laboratory, which provided a preliminary result consistent with anti-NMDAR encephalitis.

After the surgery, the patient remained hospitalized for an additional month and a half, due to not only infectious complications but also persistent episodes of confusion, cognitive slowing, headaches, and the need for motor rehabilitation. During this period, she was treated with rituximab. At discharge, the patient was cooperative and oriented, although she still experienced memory difficulties, insomnia, and motor impairments. At a follow-up appointment four months after the start of her hospitalization, she reported mild concentration difficulties but no other complaints. One year after the event, the patient was completely asymptomatic.

## Discussion

In the case described, all stages of the disease were identified in the patient, except for the prodromal phase, as reported in the literature [2]. The patient initially presented with psychiatric symptoms, which prompted her referral to the psychiatry department. It is well-documented that many patients with this condition are initially misdiagnosed with schizophrenia [7], highlighting the critical need for a meticulous differential diagnosis to distinguish between these entities.

The diagnostic delay in this case highlights the critical importance of maintaining a high index of suspicion for anti-NMDAR encephalitis, particularly in young patients presenting with compatible symptoms. Early recognition of this condition is essential to avoid unnecessary treatment delays. In this instance, the delay was partly due to the unavailability of laboratory testing for anti-NMDAR antibodies. The final diagnosis was established based on the clinical presentation, the detection of a teratoma on the second abdominopelvic CT scan (after the initial scan failed to identify it), the exclusion of other potential conditions with similar symptomatology, and, ultimately, a preliminary CSF result indicating the presence of anti-NMDAR antibodies.

This case highlights the importance of investigating teratomas in young women presenting with psychiatric or neurological symptoms suggestive of anti-NMDAR encephalitis [4]. Early imaging and a multidisciplinary approach are key to improving diagnostic accuracy and ensuring timely intervention.

## Conclusions

This case highlights the challenges of diagnosing anti-NMDAR encephalitis, particularly in young female patients presenting with acute psychiatric symptoms. The delayed identification of an ovarian teratoma and the need for extensive diagnostic testing prolonged the patient’s hospitalization, emphasizing the importance of early recognition. Despite initial setbacks, timely immunotherapy and surgical intervention led to a full recovery, reinforcing the potential for positive outcomes with appropriate treatment. A high index of suspicion and a multidisciplinary approach are crucial for optimizing patient care. Further research into early diagnostic markers is essential to improve detection and management.
